# Gut microbiota and the “gut-liver-kidney axis” theory: from mechanisms to therapeutics

**DOI:** 10.3389/fmicb.2025.1554458

**Published:** 2025-06-19

**Authors:** Dai-Liang Fei, Ying Liu, Jia Liu, Ze Yu, Jin-Liang Dong

**Affiliations:** ^1^Department of General Surgery, Zhoushan Hospital, Wenzhou Medical University, Zhoushan, Zhejiang, China; ^2^Department of Pharmacy, Zhoushan Hospital, Wenzhou Medical University, Zhoushan, China; ^3^School of Agriculture, Sun Yat-sen University, Shenzhen, Guangdong, China; ^4^The Laboratory of Cytobiology and Molecular Biology, Zhoushan Hospital, Wenzhou Medical University, Zhoushan, China

**Keywords:** gut microbiota, gut-liver-kidney axis, mechanisms, therapeutics, metabolism

## Abstract

The gut microbiota is essential for overall health, and in recent years, there has been increasing interest in its complex relationship with the “gut-liver-kidney axis.” In this research, we aim to examine how the gut microbiota affects metabolic and immune responses via influencing the functions of the liver and kidneys. It will also analyze potential pathological mechanisms involved in this interaction and discuss therapeutic strategies that focus on modulating the gut microbiota. Through thoroughly reviewing existing literature, this article intends to offer fresh perspectives and insights that could inform future research and clinical applications in this rapidly developing area of study.

## 1 Introduction

Gut microbiota is a diverse community of microorganisms that live in the gastrointestinal tract and is essential for maintaining the health of the host while influencing various bodily functions. This community includes trillions of bacteria, archaea, fungi, and viruses that interact with one another and with the host's cells, playing vital roles in digestion, metabolism, and immune system regulation. Importantly, the gut microbiota is dynamic and can change in response to several factors, including diet, age, and environmental influences, which can lead to differences in its composition. When there is dysbiosis, that is, an imbalance in the gut microbiota, it has been linked to numerous health problems, such as metabolic disorders, inflammatory diseases, and neurological conditions (Li C. et al., 2022). Additionally, gut microbiota produces metabolites like short-chain fatty acids (SCFAs), which can significantly impact the metabolism and immune responses of host, underscoring the critical role these microbial communities play in overall health (Rojas et al., 2021).

The “gut-liver-kidney axis” theory highlights the intricate relationships among the gut, liver, and kidneys, showcasing how these organs are interconnected through shared metabolic pathways and interactions with microbiota. This concept has gained attention recently as studies reveal the important roles that gut microbiota and their metabolites play in the development of liver and kidney diseases. For example, dysbiosis can lead to higher levels of harmful substances called uremic toxins in individuals with chronic kidney disease (CKD), worsening kidney function (Amini Khiabani et al., 2023). Additionally, the liver processes metabolites that originate from the gut, which can have widespread effects on kidney health, creating a feedback loop that may affect the diseases progression (Zhu et al., 2023). Understanding this axis is essential for creating targeted therapies that can adjust gut microbiota to enhance the health of both the liver and kidneys.

This article aims to explore the mechanisms that connect the gut, liver, and kidneys, as well as the implications for treatment strategies. It will examine how gut microbiota affect liver and kidney functions by producing metabolites and modulating immune responses. Furthermore, the article will highlight potential therapeutic approaches, including probiotics and dietary changes, designed to restore the balance of microbiota and enhance organ function. The structure will begin with an overview of the gut microbiota's role in maintaining health, followed by a detailed look at the gut-liver-kidney axis, and conclude with a discussion on therapeutic strategies could leverage this understanding for clinical advantages. Through integrating findings from recent studies, this work aims to shed light on the potential of microbiota-targeted therapies in managing diseases that involve the gut, liver, and kidneys (Guo et al., 2023).

## 2 Composition and function of gut microbiota

The gut microbiota, a diverse community of microorganisms living in the gastrointestinal tract, is vital for human health. This intricate ecosystem includes trillions of bacteria, viruses, fungi, and other microbes that work together to influence many bodily functions. The makeup of the gut microbiota is not static; it changes in response to various factors such as diet, age, genetics, and environmental influences. Maintaining a balanced gut microbiota is crucial for metabolic stability, immune system performance, and defense against harmful pathogens. It plays a key role in breaking down dietary fibers, producing short-chain fatty acids (SCFAs), and synthesizing essential vitamins, all of which are important for overall health. Additionally, the gut microbiota interacts with the immune system, helping to shape immune responses and preserve the integrity of the gut lining (Lukáčová et al., 2023).

### 2.1 Common types of gut microbiota and their physiological functions

The gut microbiota primarily consists of bacterial phyla, including Firmicutes, Bacteroidetes, Actinobacteria, and Proteobacteria, each of which contains various genera that perform specific functions beneficial to host. For example, Firmicutes play a significant role in fermenting dietary fibers into SCFAs, which provide energy to colon cells and possess anti-inflammatory properties. In contrast, the bacteroidetes are essential for breaking down complex carbohydrates, thereby enhancing the nutrient absorption. Furthermore, genera such as *Lactobacillus* and *Bifidobacterium*, which fall under Actinobacteria, are well-known for their probiotic effects, promoting gut health and influencing immune responses (Miles, 2020). The balance among these microbial communities is vital; for instance, an increase in Firmicutes compared to Bacteroidetes has been associated with obesity and metabolic disorders, highlighting the complex relationship between the composition of gut microbiota and the host's metabolism (Debnath et al., 2021).

### 2.2 The impact of gut microbiota dysbiosis on health

Dysbiosis refers to an imbalance in the composition of gut microbiota, which can result in negative health effects. Various factors, including a poor diet, the use of antibiotics, and chronic stress, can disturb the delicate balance of gut microbiota, leading to dysbiosis (Meng et al., 2023). This condition is often marked by a decrease in microbial diversity and an increase in harmful bacteria. Dysbiosis has been linked to several health problems, such as inflammatory bowel disease, diabetes, and cardiovascular diseases. For example, when the dysbiosis occurs, it can compromise gut barrier function, resulting in increased intestinal permeability and systemic inflammation, both of which are involved in development of various chronic diseases (Lukáčová et al., 2023). Besides, changes in the production of microbial metabolites, like SCFAs, can adversely impact the host's metabolism and immune responses, worsening health issues (Lukáčová et al., 2023). Therefore, understanding the mechanisms that contribute to gut dysbiosis is crucial for creating therapeutic approaches aimed at restoring a healthy gut microbiota and enhancing overall health outcomes (Wang et al., 2024).

In summary, the gut microbiota plays a crucial role in human health by influencing various metabolic processes, immune functions, and susceptibility to diseases. It is essential to maintain a balanced gut microbiota to prevent dysbiosis, which can lead to a range of health issues.

## 3 Physiological mechanisms of the gut-liver-kidney axis

### 3.1 Interaction between gut microbiota and the liver

Gut microbiota is essential for liver health, primarily through the gut-liver axis, which serves as a two-way communication pathway. This axis allows gut-derived metabolites and microbial products to affect liver function, while the liver can also influence the gut health. The liver is continuously exposed to substances from the gut via the portal vein, enabling the direct transport of microbial metabolites such as SCFAs, bile acids, and lipopolysaccharides (LPS), SCFAs, BAs and LPS all mediate the metabolic regulation of the gut-liver axis through the portal vein. Among them, SCFAs and BAs mainly focus on metabolic regulation and signal transmission, while LPS mainly affects liver function through the inflammatory pathway (Liu et al., 2022). These metabolites play a significant role in modulating liver metabolism and immune responses, and they can even aid in liver regeneration following injury. For example, changes in gut microbiota composition during liver regeneration indicate that these microorganisms may impact the liver's healing processes by altering the release of inflammatory factors like IL-6 and TNF-α (Xu et al., 2022).

Dysbiosis has been associated with various liver diseases, including non-alcoholic fatty liver disease (NAFLD) and hepatitis (Long et al., 2024). This relationship highlights the critical need to maintain a healthy gut microbiome to prevent liver-related health issues.

### 3.2 Role of the liver in metabolism and detoxification

Liver is the body's main metabolic organ, crucial for regulating various metabolic processes, including how the body handles fats and sugars. It detoxifies harmful substances and produces vital proteins. Maintaining metabolic balance is essential, as any disruptions can lead to serious health issues like metabolic syndrome and liver diseases (Ding et al., 2018). When the liver is injured, it often leads to metabolic problems, which can be seen in altered fat levels and insulin resistance, further worsening liver conditions (You et al., 2023). Additionally, the liver's detoxification abilities are affected by substances produced by gut bacteria. For instance, SCFAs generated during the fermentation of dietary fibers by gut microbiota can improve liver function and lower inflammation (Guo et al., 2021). Thus, understanding the liver's roles in metabolism and detoxification is vital for creating effective treatments that aim to restore liver health and prevent progression of diseases.

### 3.3 Kidney function and regulation by gut microbiota

The kidneys play a crucial role in filtering blood, maintaining fluid balance, and eliminating waste products from the body. Recent studies have shed light on the important connection between gut microbiota and kidney health, commonly referred to as the gut-kidney axis. Dysbiosis, can lead to the increased production of harmful substances called uremic toxins, which negatively impact kidney function and can accelerate the progression of chronic kidney disease (CKD) (Peters et al., 2023). For example, changes in the composition of gut microbiota have been linked to elevated levels of indoxyl sulfate and p-cresyl sulfate, both of which are known to cause kidney damage (Chen et al., 2019). Furthermore, certain metabolites produced by gut microbes can affect kidney inflammation and fibrosis, indicating that restoring a healthy gut microbiome might provide a promising new approach for treating kidney diseases (Zhu et al., 2023). This interaction between gut microbiota and kidney function highlights the critical need for a balanced microbiome to support renal health and prevent disease.

## 4 Dysbiosis of gut microbiota and associated diseases

### 4.1 Relationship between liver diseases and gut microbiota

The gut microbiota is essential for the development and progression of liver diseases, especially non-alcoholic fatty liver disease (NAFLD) and hepatitis. Dysbiosis has been associated with increased intestinal permeability. This condition allows gut-derived metabolites and pathogens to enter the bloodstream, potentially triggering liver inflammation and damage (Long et al., 2024). Research indicates that individuals with NAFLD often have altered gut microbiota profiles, which are marked by decreased microbial diversity and specific shifts in the abundance of certain bacterial groups, such as an increase in Firmicutes and a decrease in Bacteroidetes (Wree et al., 2019).

**Figure 1:**
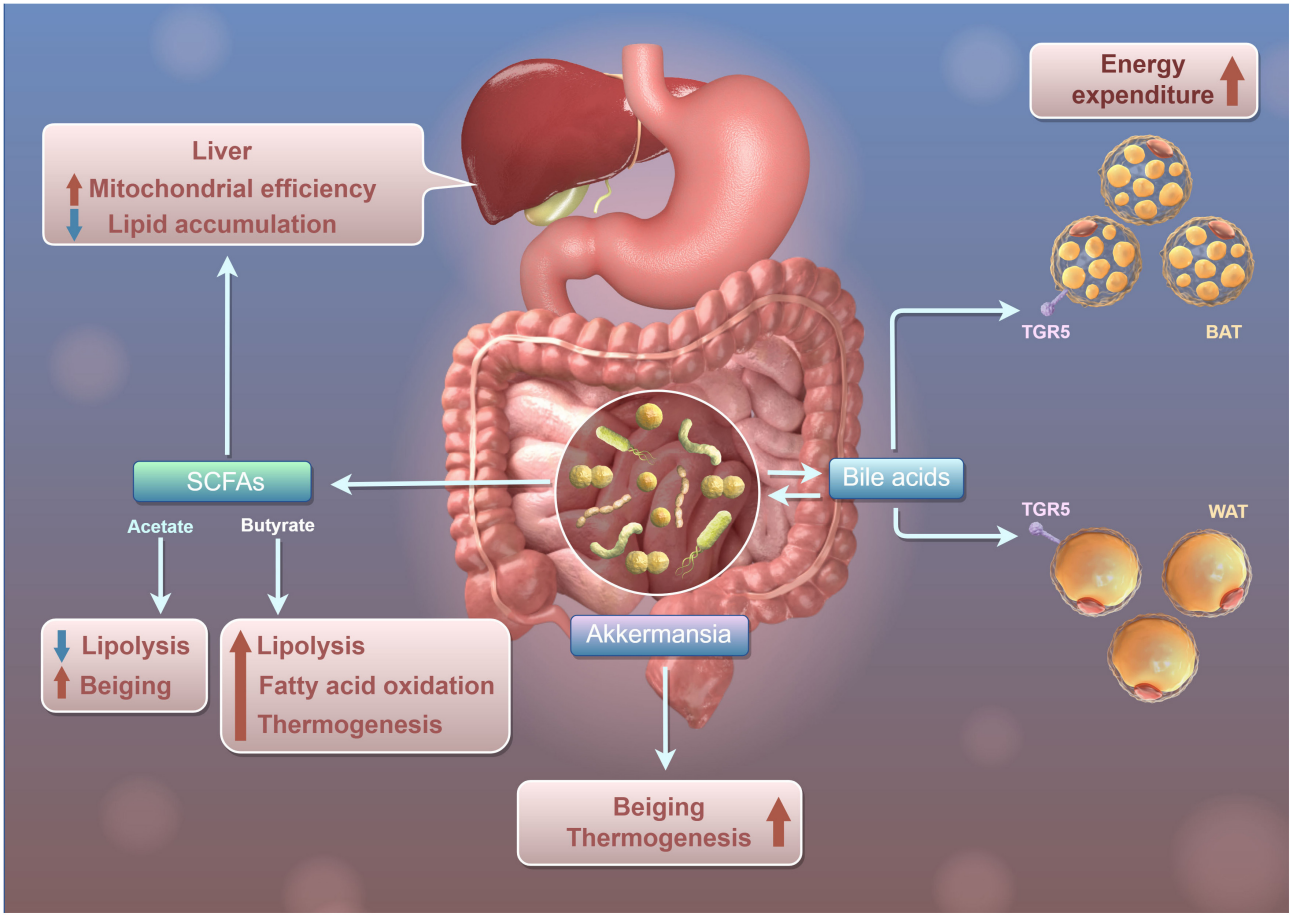
Schematic diagram of Gut Microbiota regulating the mitochondrial function and lipid metabolism of liver through SCFAs. Intestinal microbiota, SCFAs, and bile acids influence lipid metabolism and energy use in the liver and adipose tissue. The liver produces SCFAs like Acetate and Butyrate, which promote lipolysis and reduce lipid accumulation in white adipose tissue (WAT), while also enhancing fatty acid oxidation and thermogenesis for increased energy consumption. Akkermansia interacts with bile acids to boost lipolysis and fatty acid oxidation in WAT, raising heat production. Bile acids affect both WAT and brown adipose tissue (BAT) via the TGR5 receptor, increasing energy consumption in BAT and promoting lipolysis and fatty acid oxidation in WAT, thus enhancing heat production.

Additionally, the gut-liver axis serves as a vital pathway through which the gut microbiota affects liver metabolism and immune responses. For example, microbial metabolites like SCFAs and bile acids, produced in the gut, can influence hepatic lipid metabolism and inflammatory processes (Figure 1). For example, SCFAs, including propionic acid and butyric acid, can stimulate the activation of the AMPK signaling pathway by elevating the intracellular AMP/ATP ratio. The phosphorylation of AMPK leads to the inhibition of acetyl-CoA carboxylase (ACC), which subsequently lowers the levels of malonyl-CoA. This reduction alleviates the suppression of carnitine palmitoyltransferase 1 (CPT-1), thereby facilitating the transport of fatty acids into the mitochondria for β-oxidation (den Besten et al., 2015a). Additionally, SCFAs modulate the expression of uncoupling protein 2 (UCP2) via an AMPK-dependent pathway, which diminishes mitochondrial membrane potential, decreases ATP production, and further activates AMPK, thus establishing a positive feedback loop that augments oxidative metabolism (OXPHOS) (Iannucci et al., 2016). Moreover, SCFAs curtail hepatic adipogenesis by downregulating the expression and activity of PPARγ and diminishing the transcription of genes associated with lipid synthesis, such as SREBP-1c and FAS (den Besten et al., 2015b).

In the chronic liver diseases like hepatitis, the role of gut microbiota becomes increasingly significant. Dysbiosis can worsen liver inflammation through encouraging the release of pro- inflammatory cytokines and activating immune pathways (Chopyk and Grakoui, 2020). Furthermore, an experimental obesity-associated meta-flammation rat model demonstrated certain gut bacteria have been linked to the development of liver fibrosis and cirrhosis, which underscores the potential for microbiota-targeted therapies as a new method for treating liver diseases, and this discovery suggested this mechanism warrants further validation in humans (Li K. P. et al., 2022). Recent studies indicate that interventions designed to restore a healthy balance of gut microbiota, such as use of probiotics and changes in diet, may enhance liver health and slow progression of these diseases (Zhang et al., 2023). Therefore, it is crucial to understand the complex relationship between gut microbiota and liver diseases to create effective treatment strategies.

### 4.2 The impact of gut microbiota on kidney diseases

The relationship between gut microbiota and kidney diseases has received considerable attention in recent years, especially concerning chronic kidney disease (CKD) and renal failure. In patients with CKD, dysbiosis has been linked to the buildup of uremic toxins, including indoxyl sulfate and p-cresyl sulfate. These toxins can worsen kidney injury and speed up the progression of the disease (Chi et al., 2021). The gut-kidney axis is crucial in this scenario, as changes in gut microbiota can increase intestinal permeability. This alteration allows harmful metabolites to enter the bloodstream, subsequently affecting renal function (Amini Khiabani et al., 2023).

Recent research indicates that chronic kidney disease (CKD) patients often exhibit specific microbial profiles, which are marked by a reduction in beneficial bacteria and an increase in harmful species, especially hyperuricemia (HUA). A novel model of HUA in geese investigated the mechanism by which *Lactobacillus rhamnosus* GG (LGG) could have beneficial effects on HUA, LGG and its metabolites could alleviate HUA through the gut-liver-kidney axis, and LGG and proline could be promising therapies for HUA (Fu et al., 2024). These alterations can lead to an intensified inflammatory response, further advancing kidney disease. Additionally, modifying gut microbiota through dietary changes or probiotics has shown potential in lowering uremic toxin levels and enhancing kidney function (Liu et al., 2024). For example, studies have revealed that administering probiotics can improve gut barrier function and decrease inflammation, which may help slow the CKD progression (Favero et al., 2022). Therefore, focusing on gut microbiota presents a promising new strategy for treating kidney diseases and enhancing patient outcomes.

SCFAs reduce the expression of pro-inflammatory factors (such as IL-1β and MCP-1) by inhibiting the NF-κB signaling pathway. For example, in glomerular mesangial cells induced by high glucose and lipopolysaccharide, SCFAs inhibit NF-κB activation through GPR43 and reduce the generation of adhesion molecules such as ICAM-1, thereby alleviating the inflammatory response. Furthermore, SCFAs can also indirectly alleviate the damage to the kidneys caused through intestinal-derived inflammation by reducing the activity of the NLRP3 inflammasome in the colon.

### 4.3 The association between metabolic syndrome and gut microbiota

Metabolic syndrome is defined by a combination of conditions such as obesity, insulin resistance, and dyslipidemia, and it has been strongly associated with the composition and function of gut microbiota. Dysbiosis can disrupt metabolic processes, which may lead to onset of insulin resistance and obesity (Wang et al., 2022). Research indicates that people with metabolic syndrome typically have a lower diversity of gut microbiota. Specifically, there is often an increase in firmicutes and a decrease in bacteroidetes, two types of bacteria that play significant roles in how the body extracts energy from food and stores fat (Kani et al., 2023).

Gut microbiota significantly impacts metabolic health through several mechanisms, notably the production of SCFAs, which are essential in regulating appetite and glucose metabolism (Busch et al., 2024). Additionally, metabolites derived from the gut can influence systemic inflammation and insulin sensitivity, underscoring the gut's pivotal role in metabolic regulation (Guimarães et al., 2023). Recent efforts to restore the balance of gut microbiota, including the use of prebiotics, probiotics, and dietary changes, have demonstrated promise in enhancing metabolic parameters and lowering the risk of related diseases (Kani et al., 2023). Therefore, it is vital to comprehend the complex relationship between gut microbiota and metabolic syndrome to develop the targeted therapies aimed at addressing this escalating health concern.

## 5 Therapeutic strategies for modulating gut microbiota

### 5.1 Gut microbiota regulation treatment strategies

Gut microbiota is essential for maintaining host health and can be significantly influenced by different therapeutic approaches. These strategies focus on restoring or maintaining a balanced gut microbiota, which is vital for optimal physiological functions and the prevention of diseases. This article will delve into the applications of probiotics and prebiotics, dietary interventions, and the potential effectiveness of microbiota transplantation.

### 5.2 Application of probiotics and prebiotics

Probiotics and prebiotics play crucial roles in shaping gut microbiota. Probiotics are live microorganisms that provide health benefits when taken in sufficient quantities, while prebiotics are non-digestible food components that encourage the growth and activity of beneficial gut bacteria. Recent study has underscored the importance of probiotics in preventing and managing various gastrointestinal issues, such as irritable bowel syndrome and inflammatory bowel disease (So et al., 2023). Additionally, probiotics may enhance immune responses and lower the risk of infections (Rojas et al., 2021). Prebiotics, including substances like inulin and fructooligosaccharides, act as food sources for beneficial bacteria, leading to the production of SCFAs that possess anti-inflammatory effects. The combined effects of probiotics and prebiotics, often termed synbiotics, can further improve gut health by fostering a balanced microbiota and enhancing metabolic functions (Markowiak and Sliżewska, 2017). However, the effectiveness of these interventions can differ based on individual microbiota profiles, dietary patterns, and overall health, highlighting the need for personalized therapeutic approaches (Li et al., 2021).

**Figure 2:**
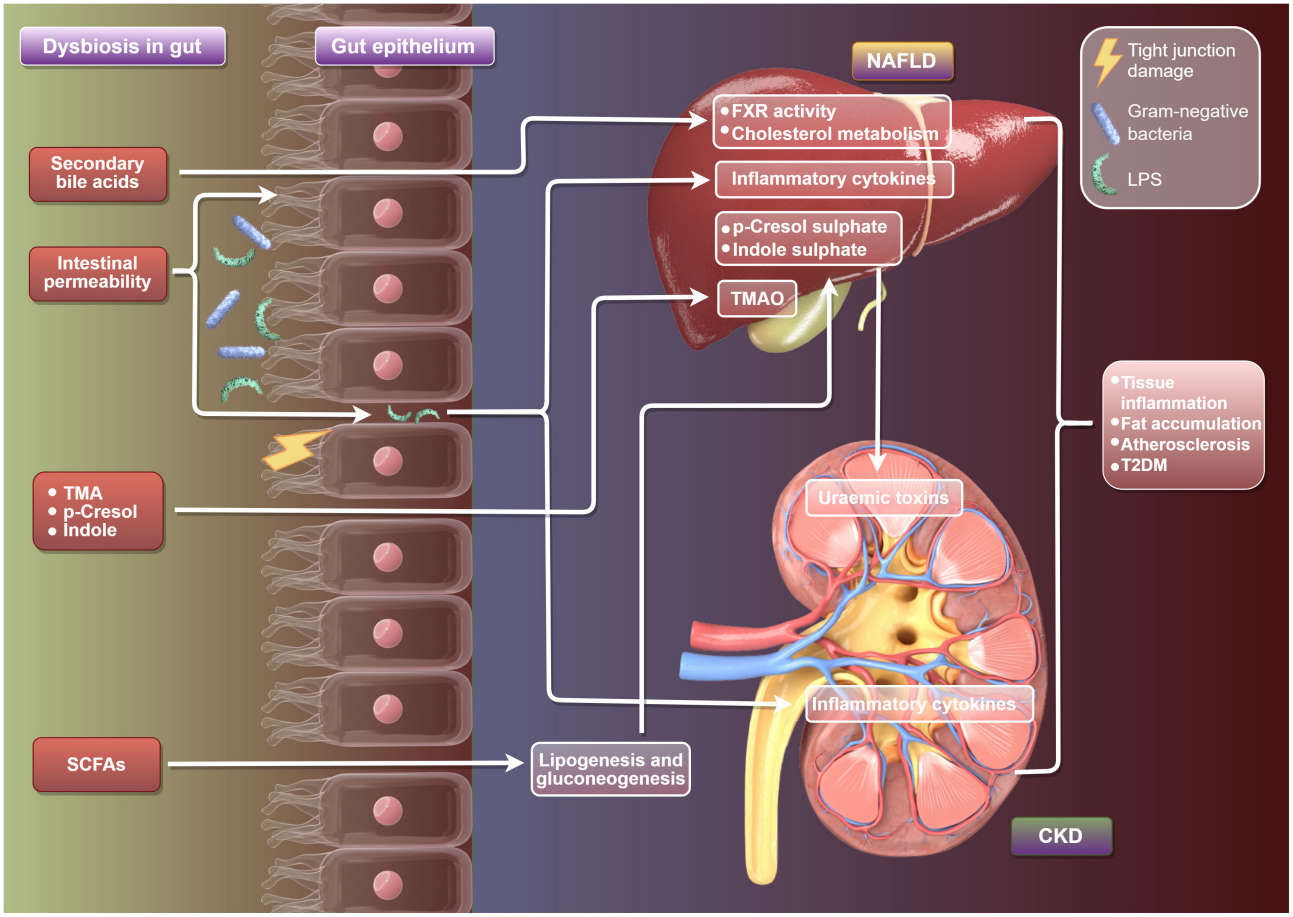
Schematic diagram of the mechanism by which gut microbiota regulate the development of NAFLD and CKD. It illustrates the link between gut dysbiosis and NAFLD and CKD, highlighting gut bacteria imbalance, secondary bile acids, TMA, SCFAs, and their effects on intestinal cells, leading to inflammation and metabolic issues in NAFLD and CKD, including increased risks of inflammation, fat accumulation, and T2DM.

At present, the clinical research evidence regarding the prognostic impact of prebiotics or probiotics on patients with non-alcoholic fatty liver disease (NAFLD) or chronic kidney disease (CKD) is still limited (Figure 2). Firstly, some small-scale clinical trials have observed that probiotics may improve liver enzyme (ALT/AST) levels, liver fat content or inflammatory markers. However, most of these studies are short-term interventions (usually 3–6 months), lacking long-term follow-up data for hard endpoints such as liver fibrosis progression, liver cirrhosis or liver cancer. In addition, studies using compound strains containing *Bifidobacterium* and *Lactobacillus* have shown an improvement effect on liver steatosis, but these results have not yet been verified in large-scale multicenter RCTS (Carpi et al., 2022). Secondly, some studies on CKD patients mainly focus on the regulatory effect of probiotics on uremic toxins (such as cresol and indolethol sulfate), which are closely related to the metabolism of the intestinal flora. Some studies have shown that probiotics may reduce toxin levels, but there is currently no evidence to suggest that this effect can translate into delaying renal function decline or reducing the risk of dialysis. Studies on hemodialysis patients have found that probiotics may improve the micro-inflammatory state, but no significant impact on cardiovascular events or survival rate has been observed (Cooper et al., 2023).

### 5.3 Impact of dietary interventions on gut microbiota

Diet plays a crucial role in shaping the composition and function of gut microbiota, which can be modified through various dietary choices. Research indicates that different dietary patterns, such as high-fiber, Mediterranean, and plant-based diets, can enhance the diversity and stability of gut microbiota (Chen et al., 2024). A key component of this is dietary fiber, which gut bacteria ferment to produce SCFAs. SCFAs are essential for maintaining gut health and managing inflammation (Fu et al., 2022). In contrast, diets that are high in saturated fats and refined sugars can disrupt this balance, leading to dysbiosis, which is marked by a decrease in microbial diversity and an increase in harmful bacteria, an experiment from a mice model shows that FMT from different donors coupled with dietary fiber intervention could lead to different patterns of gut microbiota composition, and dietary fiber might play a more critical role in shaping gut microbiota than FMT donor (Zhong et al., 2021). Notably, long-term dietary changes tend to result in more stable modifications to gut microbiota than short-term dietary adjustments, highlighting the importance of consistent dietary habits for maintaining microbiota health (Leeming et al., 2019).

Besides, there is growing evidence that gut microbiota can affect how dietary components are metabolized, which in turn influences the host's metabolism and the likelihood of developing chronic conditions such as obesity and type II diabetes (Li et al., 2021). Therefore, dietary interventions hold significant potential as a strategy for modifying gut microbiota and enhancing overall health outcomes.

### 5.4 Potential efficacy of microbiota transplantation

Fecal microbiota transplantation (FMT) has emerged as a promising treatment for restoring the balance of gut microbiota, especially in cases of dysbiosis linked to recurrent Clostridioides difficile infections and various gastrointestinal disorders. The procedure involves transferring fecal matter from a healthy donor to a recipient, with the goal of re-establishing a diverse and functional microbiota. Clinical studies have shown that FMT can lead to sustained remission in patients suffering from recurrent infections and can significantly improve overall gut health (Halaweish et al., 2022). However, the success of FMT is influenced by several factors, including the composition of the donor's microbiota, the health status of the recipient, and the method used for the transplantation (Porcari et al., 2023). While FMT holds promise for treating specific conditions, its potential applications in broader therapeutic areas, such as metabolic diseases and neurodegenerative disorders, are still being researched. As studies continue to investigate the safety and effectiveness of FMT, this approach may open doors to innovative microbiota- targeted therapies that could enhance health and prevent disease.

In brief, the modulation of gut microbiota using probiotics, prebiotics, dietary changes, and fecal microbiota transplantation offers a comprehensive strategy for enhancing health outcomes. Ongoing research is crucial to refine these approaches and to gain a deeper understanding of how they function within the intricate ecosystem of the human gut.

## 6 Future research directions and clinical application prospects

### 6.1 Application of emerging technologies in gut microbiota research

Emerging technologies are transforming the study of gut microbiota, offering deeper insights into its intricate interactions with host physiology and health. Techniques like metagenomics, metatranscriptomics, and metaproteomics enable researchers to examine microbial communities with unprecedented detail. For example, high-throughput sequencing technologies allow for the identification and quantification of various microbial species in the gut, which helps in exploring their functional roles in both health and disease (Sahle et al., 2024). Moreover, advancements in organ-on-a-chip models and gut-on-a-chip systems are improving our understanding of how the gut microbiome responds to different stimuli, such as dietary changes and pharmacological treatments (Thomas et al., 2023). These technologies enhance our capability to study microbial diversity and function while also paving the way for the personalized medicine, where treatments can be customized according to an individual's specific microbiota composition. The incorporation of artificial intelligence (AI) in analyzing microbiome data further simplifies the identification of patterns and the prediction of health outcomes, potentially leading to new therapeutic strategies aimed at addressing gut microbiota dysbiosis (Shen et al., 2025). Overall, the use of these cutting-edge technologies holds great promise for deepening our understanding of gut microbiota and its significant implications for human health.

### 6.2 Integration of personalized medicine with gut microbiota

The intersection of personalized medicine and gut microbiota research is revolutionizing healthcare through recognizing how individual differences in gut microbiota composition can significantly affect drug metabolism, efficacy, and toxicity. This underscores the necessity for personalized treatment strategies (Feng et al., 2020). For instance, research has demonstrated gut microbiome can influence how various medications are processed in the body, resulting in varying therapeutic outcomes for different patients (Lim and Wang, 2022). Through utilizing this information, healthcare providers can customize drug therapies according to a patient's unique microbiome profile, which can enhance treatment effectiveness while reducing the risk of adverse effects.

Additionally, advancements in next-generation probiotics and other therapies aimed at targeting the microbiota are being developed to restore microbial balance and improve health outcomes in a personalized way (Singh and Natraj, 2021). The potential for incorporating gut microbiota profiling into clinical practice is extensive, as it can guide dietary recommendations, inform probiotic usage, and assist in selecting appropriate pharmacological agents. This personalized approach not only improves patient care but also deepens our understanding of the complex relationship between the gut microbiome and overall health.

### 6.3 Role of policy and education in gut health

Promoting gut health through effective policy and education is essential for tackling the increasing prevalence of health issues related to the microbiota. Public health initiatives that emphasize nutrition education, dietary guidelines, and the significance of gut microbiota can empower individuals to make informed choices that enhance their gut health (Zhang et al., 2023). For example, educational programs that showcase the advantages of a diverse diet, particularly one rich in fiber and fermented foods, can motivate people to adopt healthier eating habits, which can positively affect the composition of their gut microbiota (Li C. et al., 2022).

Furthermore, policies that advocate for research funding and the incorporation of microbiome studies into healthcare practices can expedite progress in this area. It is crucial for governments and health organizations to prioritize the creation of guidelines that endorse microbiota-friendly practices, such as recommending the inclusion of probiotics and prebiotics in diets. Additionally, fostering collaboration among researchers, healthcare professionals, and policymakers can help translate scientific discoveries into practical health policies. By increasing public awareness and understanding of gut health, we can lay the groundwork for better health outcomes and alleviate the burden of diseases associated with microbiota.

## 7 Conclusions

The intricate relationship between the gut microbiome and the gut-liver-kidney axis has gained significant attention in recent years, highlighting the complex interactions within this triad. The gut microbiome, a diverse ecosystem of microorganisms residing in the gastrointestinal tract, plays a vital role in various metabolic processes, immune function, and overall health. Its influence reaches liver and kidneys, where microbial metabolites can affect systemic inflammation, contribute to metabolic syndrome, and even play a role in chronic kidney disease.

Understanding the relationship between the gut microbiome and health is crucial for developing therapeutic strategies that aim to modify the gut microbiome to improve patient outcomes across a range of diseases. Recent research emphasizes the promise of probiotics, prebiotics, and dietary changes in restoring microbial balance and enhancing the functionality of the gut-liver-kidney axis. Nevertheless, a significant challenge remains in reconciling the varied findings from different studies, as many report conflicting results. These discrepancies often arise from differences in study design, population characteristics, and methodologies employed, making it difficult to draw definitive conclusions.

As experts in the field, advocating for standardized research protocols is crucial to enable the comparison of outcomes across various studies. Future research should aim not only to clarify the specific mechanisms through which the gut microbiome affects liver and kidney function but also to investigate the therapeutic implications of these interactions. Longitudinal studies that track the evolution of the gut microbiome over time in relation to liver and kidney health will be especially valuable.

In addition to indirect liver-kidney connection mediated by enterogenic factors, hepatogenic factors are also an important factor. For instance, it is common that the intestinal barrier dysfunction in patients with liver cirrhosis can aggravate renal dysfunction. The reason for this clinical phenomenon is that portal hypertension in liver cirrhosis leads to intestinal mucosal congestion and edema, and tight junction proteins form “leakage of the intestine.” At this point, lipopolysaccharide (LPS) released by Gram-negative bacteria in the intestine enters the portal vein circulation through the damaged barrier. The Kupffer cells in the hardened liver are impaired in function and unable to effectively clear LPS, resulting in systemic endotoxemia. Then, the LPS that has not been degraded by the liver activates the TLR4 receptors in the kidneys. It induces apoptosis of renal tubular epithelial cells (through the caspase-3 pathway). endotoxin stimulates excessive production of NO throughout the body, triggers renal artery constriction and renal cortical ischemia, and induces typical manifestations of hepatorenal syndrome (HRS) (Bera and Wong, 2022).

The clinical implications of these findings are significant and warrant attention. As we enhance our understanding of the gut-liver-kidney axis, it becomes increasingly important to apply this knowledge in clinical settings. This involves creating targeted interventions that utilize gut microbiome modulation to either prevent or treat diseases impacting the liver and kidneys.

By adopting personalized medicine approaches that take into account an individual's unique microbiome profile, we could transform treatment methodologies, resulting in more effective and customized therapeutic strategies.

Meanwhile, we also pay attention to the differences and limitations between animal models and human studies in the research on the association between gut microbiota and liver diseases. The main points are as follows:

1. Physiological and metabolic differences: there are notable differences in bacterial genera proportions and immune responses between rats and humans; for example, *Lactobacillus* makes up over 50% in rats' cecal microbiota but <5% in humans, and rodents' immune systems are more sensitive to bacterial products, causing heightened inflammatory responses;
2. Simplification of disease modelconstruction: experimental rat models quickly develop liver fibrosis in weeks, while humans take 10–20 years for NASH to progress to cirrhosis due to factors like obesity and insulin resistance;
3. Clinical transformation bottleneck: animal studies use histological improvements, while human trials focus on hard endpoints, but their correlation is unclear. Rats regenerate liver tissue much faster than humans, masking fibrosis risk; for instance, rats can regenerate 70% of their liver in 7 days, while humans take months with fibrosis risk.

In conclusion, it is essential to bridge the gap between research and clinical application to fully utilize the therapeutic potential of the gut microbiome. The intricate relationships within the gut-liver-kidney axis require ongoing exploration, emphasizing the importance of integrating various research perspectives to enhance clinical practice. As we deepen our understanding, we must prioritize collaboration across different disciplines to discover new opportunities in patient care, ultimately leading to better health outcomes for individuals affected by disorders related to this crucial axis.

Based on the above summary, we propose the “Microbial metabolic cycle—Organ Barrier Interaction” hypothesis, suggesting that intestinal microbiota influences the liver and kidneys through nutritional and toxin metabolic cycles, with the intestinal barrier's integrity being crucial.

1. Positive cycle: SCFAs may enhance TLR4 sensitivity in kidneys through liver epigenetic changes, promoting immune tolerance.
2. Negative cycle: intestinal barrier damage allows endotoxins and metabolites into circulation, overloading liver enzymes and harming kidneys, increasing oxidative stress.
3. Interaction hubs: the intestinal barrier, hepatic endothelial structure, and glomerular filtration barrier form a three-tier defense system.

Key unexplored areas include:

1. The spatiotemporal dynamics of metabolic flows among the gut, liver, and kidney, particularly the role of liver-synthesized apoA-IV in bile acid metabolism via renal feedback, which lacks quantitative tracer evidence.
2. The organ-specific colonization of microbial communities, where bacterial fragments may enter systemic circulation through liver Kupffer cells, forming microcolonies in kidneys and causing inflammation.
3. Cross-border regulation of the neuro-endocrine axis, with intestinal microbiota metabolites potentially coordinating anti-inflammatory responses in the liver and kidneys via the α7nAChR receptor, which remains unverified.

Future research should focus on the monitoring technology for the three-organ linkage, developing microbiota intervention strategies based on metabolic simulations, and validating a treatment paradigm that prioritizes intestinal barrier repair, liver metabolism rebalancing, and renal excretion optimization.
